# Cervical intraepithelial neoplasia progression and regression among women living with HIV in Zambia

**DOI:** 10.1111/hiv.70134

**Published:** 2025-11-05

**Authors:** John Andoh, Katayoun Taghavi, Misinzo Moono, Partha Basu, Thamsanqa Madliwa, Mulindi H. Mwanahamuntu, Nicola Low, Albert Manasyan, Eliane Rohner

**Affiliations:** ^1^ Institute of Social and Preventive Medicine University of Bern Bern Switzerland; ^2^ Early Detection, Prevention and Infections Branch International Agency for Research on Cancer, World Health Organization (IARC/WHO) Lyon France; ^3^ Centre for Infectious Disease Research in Zambia Lusaka Zambia; ^4^ Lancet Laboratories Johannesburg South Africa; ^5^ Women and Newborn Hospital University Teaching Hospitals Lusaka Zambia; ^6^ University of Alabama at Birmingham Birmingham Alabama USA

**Keywords:** cervical intraepithelial neoplasia, high‐risk HPV, HIV, precancer treatment, Zambia

## Abstract

**Objectives:**

Cervical screening and precancer treatment are less effective in women living with HIV (WLWH) than in women without HIV. We assessed high‐risk human papillomavirus (HR‐HPV) infection and cervical disease progression among screened WLWH in Zambia.

**Methods:**

Participants underwent visual inspection with acetic acid (VIA), HR‐HPV testing and cervical biopsies at baseline and at follow‐up 30–36 months later. Women with positive VIA results or high‐grade histology were offered treatment. We assessed HR‐HPV and cervical disease prevalence at both timepoints and used multivariable logistic regression to identify factors associated with cervical disease progression and regression.

**Results:**

Among 241 included women, HR‐HPV prevalence declined from 44% (95% confidence interval [CI]: 39%–49%) at baseline to 24% (95% CI: 19%–31%) at follow‐up. High‐grade disease decreased from 25% (95% CI: 20%–31%) to 9% (95% CI: 5%–13%). In analyses adjusted for age, CD4 cell count, HIV RNA viral load, HR‐HPV infection and histological results at baseline, precancer treatment was associated with increased odds of disease regression (adjusted odds ratio [aOR]: 2.74, 95% CI: 1.08–7.06) and reduced odds of progression (aOR: 0.45, 95% CI: 0.11–1.64). One‐third of women with high‐grade disease at follow‐up (7/21) had previously undergone precancer treatment.

**Conclusions:**

Cervical screening and precancer treatment are key to reducing cervical disease progression among WLWH and ultimately achieving cervical cancer elimination, but efforts to improve treatment effectiveness among WLWH must be balanced with the risk of overtreatment.

## INTRODUCTION

Cervical cancer incidence rates are highest in sub‐Saharan Africa [1], a region with a high HIV prevalence [2]. Persistent infection with high‐risk human papillomavirus (HR‐HPV) genotypes is a necessary cause of virtually all cervical cancers [3]. Women living with HIV (WLWH) have a higher risk of acquiring HR‐HPV and are more likely to experience persistent HR‐HPV infection [4]. Furthermore, WLWH have an approximately six times greater risk of developing cervical cancer than women without HIV [4]. The increased risk of HR‐HPV infection and cervical disease among WLWH is due to overlapping behavioural risk factors and biological mechanisms such as HIV‐related immunodeficiency [5].

Zambia has the second‐highest cervical cancer incidence worldwide (age‐standardized rate: 72/100000 women) [1], and a high HIV prevalence (12.6% among women aged 15–49 years) [6]. While cervical cancer screening programmes have reduced the cervical cancer burden in high‐income countries, limited resources and a high HIV prevalence make it difficult for countries like Zambia to replicate this [7]. Zambia's screening programme, until recently, relied on visual inspection with acetic acid (VIA) [8], a test with limited accuracy [9], particularly among WLWH [10]. In contrast, HR‐HPV tests have a high sensitivity for the detection of cervical intraepithelial neoplasia grade 2 or higher (CIN2+). Thus, the 2021 World Health Organization (WHO) cervical cancer screening guidelines strongly suggest HR‐HPV testing as the primary screening method and advise that VIA‐based screening programmes transition rapidly [11]. In 2023, Zambia updated its screening guidelines and started scaling up HR‐HPV testing as the primary screening test for all women, including WLWH [11]. However, several challenges regarding the prevention of cervical cancer among WLWH remain. For example, the WHO recommends a screen, triage and treatment approach for HR‐HPV‐positive WLWH, but the optimal triage test remains unclear. Furthermore, persistence and recurrence of cervical disease after cervical precancer treatment are high among WLWH [12].

We aimed to describe changes in HR‐HPV prevalence and identify factors associated with cervical disease progression and regression between a baseline and a follow‐up visit 30–36 months later among screened WLWH in Zambia to inform cervical cancer prevention in this population.

## MATERIALS AND METHODS

### Study design and participants

We conducted a cohort study among WLWH who had participated in a screening test accuracy study at Kanyama Hospital, Lusaka, Zambia, described in detail elsewhere [13]. Briefly, 375 WLWH aged 18–65 years who attended the clinic and met the eligibility criteria during the recruitment period were consecutively enrolled in the parent study [14]. Women with a history of cervical cancer, a total hysterectomy, or HPV vaccination were ineligible. We attempted to contact all 375 included women and invited them for a follow‐up visit after 30–36 months.

### Study procedures

Women who consented to take part in the follow‐up study underwent the same procedures as at their first (baseline) visit. At both visits, a study nurse collected blood samples to measure CD4 cell counts and HIV RNA viral loads. A trained study nurse performed a speculum examination, collected a cervical sample using a cyto‐broom and performed VIA. After collection, the cyto‐broom was immediately placed into ThinPrep PreservCyt solution (Hologic, Marlborough, Massachusetts, USA). A research assistant used the GeneXpert platform (Cepheid, Sunnyvale, California, USA) to test the cervical samples for HR‐HPV. The GeneXpert HPV test detects 14 HR‐HPV genotypes (HPV 16, 18, 45, 31, 33, 35, 39, 51, 52, 56, 58, 59, 66 and 68). The study nurse took cervical biopsies from all participants. If visual cervical lesions were present, the study nurse took at least two biopsies from the most severe lesions. If no visible lesions were present, the study nurse obtained one biopsy from each quadrant. The cervical biopsies were histologically assessed using the lower anogenital squamous terminology (LAST). If a sample showed CIN2 or ambiguous findings, p16 immunostaining was used [15]. At baseline, slides were assessed at a South African laboratory with the capacity for p16 staining, then sent to a Zambian laboratory for verification. Consensus was reached via joint teleconference review. Histological assessment by the South African laboratory was used for clinical decisions to ensure timely treatment, and consensus diagnosis was used in the analysis. At follow‐up, the histological assessment was done at the South African laboratory only. Data were collected using paper case report forms. A trained data associate entered the data into a password‐protected electronic database (Research Electronic Data Capture, REDCap, Vanderbilt University, Nashville, USA).

### Treatment

All women with VIA‐positive results or with high‐grade squamous intraepithelial lesion [HSIL]‐CIN2+ on histological assessment by the South African laboratory were offered cervical precancer treatment with cryotherapy, thermal ablation, or loop electrosurgical excision procedure (LEEP), as clinically indicated. Women who did not return for cervical precancer treatment were first contacted by phone. Those who could not be reached or still did not attend were then traced by a community health worker. All participants received up to three phone invitations and one in‐person follow‐up invitation. At the follow‐up visit, we asked participants about treatment for cervical precancer that they may have received outside of the study.

### Inclusion criteria and definitions

We included all women who returned for the follow‐up visit and had cervical histology results available at both timepoints. In line with the LAST classification, we classified the cervical disease state into four categories based on the consensus histological report from two pathologists at baseline or the histology report from a single pathologist at follow‐up: normal (no histological abnormalities), low‐grade disease (CIN1 or low‐grade squamous intraepithelial lesion [LSIL]‐CIN2), high‐grade disease (HSIL‐CIN2 or HSIL‐CIN3) and cervical cancer. In consensus, some histological diagnoses were upgraded to HSIL‐CIN2 despite the absence of p16 results. Results for p16 staining were mainly missing if the pathologist at the laboratory where p16 staining was performed had categorized a specific sample as normal or LSIL on independent review, but the sample was later upgraded to HSIL by consensus. In a sensitivity analysis, we reclassified HSIL‐CIN2 without p16 staining as low‐grade disease.

We defined outcomes using the histological results at baseline and the follow‐up visit. We defined cervical disease regression as: (1) low‐grade disease at baseline followed by normal histology, or (2) high‐grade disease at baseline followed by low‐grade or normal histology. Cervical disease progression: (1) normal histology at baseline followed by low‐grade, high‐grade, or cancerous disease, or (2) low‐grade disease at baseline followed by high‐grade or cancerous disease or (3) high‐grade disease followed by cancerous disease. We defined persistence as the same cervical disease state at both timepoints.

### Statistical analysis

We used descriptive statistics to examine the characteristics of the study population. We calculated the HR‐HPV prevalence with 95% confidence intervals (CIs) at both timepoints. We applied two binary logistic regression models to determine factors potentially associated with disease progression or regression. The first model compared progression against no progression (persistence or regression) among women with normal or low‐grade cervical disease at baseline. The second model compared regression against no regression (persistence or progression) among women with low‐grade or high‐grade cervical disease at baseline. We performed univariable and multivariable logistic regression analyses to examine the following baseline explanatory variables: age (continuous variable), CD4 cell count (cells/μL; continuous variable), HIV RNA viral load (copies/mL; categorized as detectable or undetectable, with <50 copies/mL deemed undetectable), HR‐HPV infection (positive/negative) and cervical disease status (normal, low‐grade and high‐grade). We also included any pre‐cancer treatment administered during the study (yes/no). Furthermore, we considered HR‐HPV infection dynamics from baseline to follow‐up for women who did not undergo cervical precancer treatment. The HR‐HPV dynamics were categorized as persistently negative (negative for all HR‐HPV at both timepoints), new infection (negative at baseline and positive for any HR‐HPV at follow‐up), cleared infection (positive for any HR‐HPV at baseline and negative for all HR‐HPV at follow‐up) and persistently positive (positive for any HR‐HPV at both timepoints) in women without cervical precancer treatment. A priori knowledge initially informed the model selection, and likelihood ratio tests based on the Bayesian information criteria (BIC) further guided it. We conducted global likelihood ratio tests to draw comparisons between similar models. We used R version 4.2.2 (R Foundation for Statistical Computing, Vienna, Austria) for the analyses.

## RESULTS

### Participants

Of 375 women who participated in the screening test accuracy study, 247 returned for a follow‐up visit. The median time to follow‐up was 34 months. Of 128 women who did not return for the follow‐up visit, 79 had changed their phone number or address and could not be reached. Other reasons are detailed in Figure S1. Of note, four women had died, with one death attributed to cervical cancer. Of 247 women who returned for the follow‐up visit, 241 women had complete cervical histology results at both timepoints available and were included in the analysis.

At the baseline visit, the median age of the included women was 38 years (interquartile range [IQR] 32–45; Table 1). Most women (81%) had lower than a secondary level of education, and about half (51%, *n* = 122) reported 2–3 lifetime sexual partners. At baseline, all women were on antiretroviral therapy (ART), and most of them had well‐controlled HIV disease, with a median CD4 cell count of 561 cells/μL (IQR 423–770) and 88% with undetectable HIV RNA viral loads.

**TABLE 1:** Demographic characteristics of included women living with HIV at baseline.

	Overall *N* = 241
Median age (IQR) [years]	38 (32, 45)
Age group	
18–29 years	44 (18%)
30–39 years	97 (40%)
≥40 years	100 (41%)
Educational level	
<Secondary	196 (81%)
≥Secondary	45 (19%)
Lifetime sexual partners	
1	38 (16%)
2–3	122 (51%)
≥4	81 (34%)
Median age of sexual debut (IQR) [years]	17 (16, 19)
Median time since HIV diagnosis (IQR) [years]	5.5 (3.0, 11.0)
Missing	17
Median time since ART initiation (IQR) [years]	5.0 (3.0, 9.8)
Missing	15
Median CD4 cell count (IQR) [cells/μL]	561 (423, 770)
Detectable HIV RNA viral load	28 (12%)
Cervical precancer treatment during study period	61 (25%)

Abbreviations: ART, antiretroviral therapy, IQR, interquartile range.

### Cervical precancer treatment

During the study period, 82 women required treatment for precancerous cervical lesions based on VIA or histology results, of whom 61 (74%) underwent treatment. Three women received repeat cervical precancer treatment during the study period for persistent/progressive disease. About half of the 58 women who were treated once (*n* = 30, 52%) had high‐grade disease on the baseline consensus histology result (13 VIA‐positive, 17 VIA‐negative); the other 28 women had no evidence of high‐grade disease on consensus histology (14 VIA‐positive, 14 VIA‐negative). Among the 14 women who were VIA‐negative and had no high‐grade disease on the consensus histology assessment, 4 were treated for high‐grade disease reported on the initial histological assessment (before a consensus diagnosis was reached), 5 were treated based on persistent low‐grade disease in the presence of HR‐HPV and 5 were treated outside of the study. Of the 58 women with a single treatment, 36 women (62%) received excisional and 22 (38%) ablative treatments. Among the three women who underwent repeat treatments, two received cryotherapy followed by LEEP, while one woman underwent repeat LEEPs.

### HR‐HPV and cervical disease prevalence over time

**FIGURE 1:**
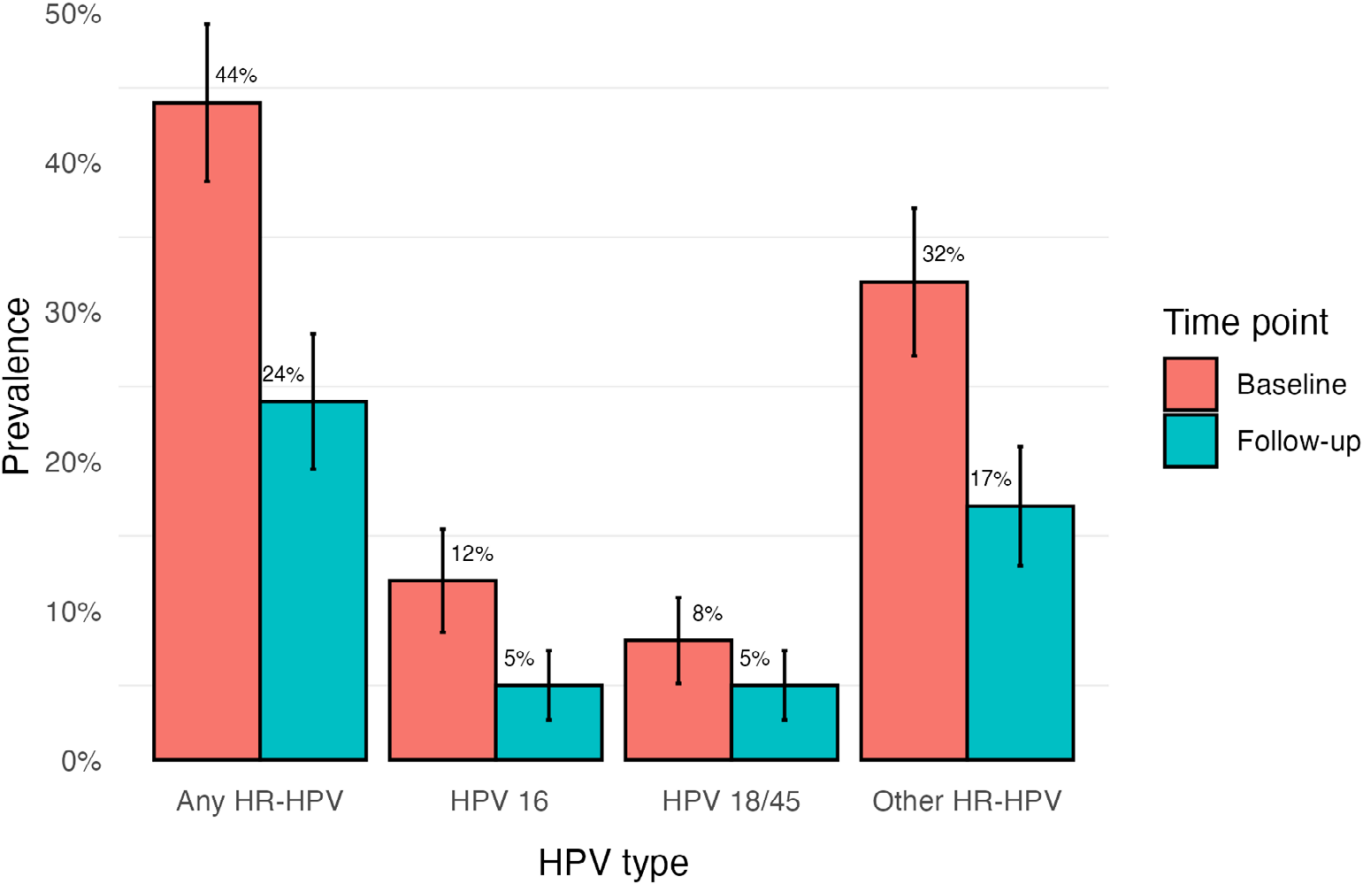
High‐risk human papillomavirus (HR‐HPV) prevalence at the baseline and follow‐up visit.

The prevalence of high‐grade disease decreased from 25% (*n* = 61) at baseline to 9% (*n* = 21) at follow‐up, while the prevalence of low‐grade disease increased from 51% (*n* = 122) to 70% (*n* = 169). Concurrently, the prevalence of any HR‐HPV decreased from 44% (95% CI: 39%–49%) at baseline to 24% (95% CI: 19%–31%) at follow‐up (Figure 1). Similarly, HPV 16 prevalence decreased from 12% (95% CI: 8%–16%) to 5% (95% CI: 2%–8%), and HPV 18/45 prevalence dropped from 8% (95% CI: 5%–11%) to 5% (95% CI: 2%–8%).

### Factors associated with disease progression and regression

Table 2 presents unadjusted and adjusted odds ratios (OR) for factors potentially associated with cervical disease progression and regression. HR‐HPV infection at baseline was associated with disease progression when adjusting for age, CD4 cell count, HIV RNA viral load, cervical histology at baseline and cervical precancer treatment (adjusted OR: 3.51, 95% CI: 1.26–11.1). Women with normal histology results had substantially higher odds of disease progression than those with low‐grade disease (adjusted OR: 47.2, 95% CI: 17.7–150.6). Cervical precancer treatment tended to be associated with reduced odds of disease progression (adjusted OR: 0.45, 95% CI: 0.11–1.64) and approximately 3‐fold higher odds of disease regression (adjusted OR: 2.74, 95% CI: 1.08–7.06). Disease regression was more common among women with high‐grade than low‐grade disease (adjusted OR: 17.82, 95% CI: 7.85–44.8) and less likely among women with an HR‐HPV infection at baseline (adjusted OR: 0.45, 95% CI: 0.18–1.05). The sensitivity analysis reclassifying HSIL‐CIN2 without p16 staining as low‐grade disease yielded similar results (Table S2).

**TABLE 2:** Crude and adjusted odds ratios (ORs) for factors potentially associated with cervical disease progression and regression (adjusting for age, CD4 cell count, HIV RNA viral load, high‐risk HPV, cervical disease at baseline and precancer treatment).

	Progression among 180 women with normal or low‐grade cervical disease at baseline				Regression among 183 women with low‐grade or high‐grade cervical disease at baseline			
	Progression (*N* = 52)	No progression (*N* = 128)	OR (95% CI)	aOR (95% CI)	Regression (*N* = 79)	No regression (*N* = 104)	OR (95% CI)	aOR (95% CI)
Age (per 10‐year increase)	‐	‐	1.11 (0.76, 1.64)	1.41 (0.82, 2.48)	‐	‐	0.84 (0.61, 1.16)	0.77 (0.51, 1.15)
HIV CD4 (per 100 increase)	‐	‐	1.08 (0.95, 1.22)	1.16 (0.98, 1.38)	‐	‐	0.97 (0.86, 1.08)	0.96 (0.82, 1.12)
HIV RNA viral load								
Undetectable	45 (87%)	116 (91%)	Ref	Ref	70 (89%)	91 (88%)	Ref	Ref
Detectable	7 (13%)	12 (9%)	1.50 (0.53, 3.99)	2.99 (0.73, 12.71)	9 (11%)	13 (13%)	0.90 (0.35, 2.21)	0.44 (0.11, 1.54)
High‐risk HPV infection at baseline								
No	32 (62%)	79 (62%)	Ref	Ref	38 (48%)	54 (52%)	Ref	Ref
Yes	20 (38%)	49 (38%)	1.01 (0.51, 1.95)	3.51 (1.26, 11.11)	41 (52%)	50 (48%)	1.17 (0.65, 2.09)	0.45 (0.18, 1.05)
Cervical histology at baseline								
Normal	42 (81%)	16 (13%)	29.4 (12.9, 73.4)	47.2 (17.7, 150.6)	‐	‐	‐	‐
Low‐grade disease	10 (19%)	112 (88%)	Ref	Ref	28 (35%)	94 (90%)	Ref	Ref
High‐grade disease	‐	‐	‐	‐	51 (65%)	10 (10%)	17.1 (7.99, 39.9)	17.82 (7.85, 44.8)
Precancer treatment								
No	46 (88%)	103 (80%)	Ref	Ref	45 (57%)	84 (81%)	Ref	Ref
Yes	6 (12%)	25 (20%)	0.54 (0.19, 1.32)	0.45 (0.11, 1.64)	34 (43%)	20 (19%)	3.17 (1.65, 6.23)	2.74 (1.08, 7.06)

Abbreviations: aOR, adjusted odds ratio; CI, confidence interval; HPV, human papillomavirus.

When we restricted the analysis to women who did not undergo cervical precancer treatment, women with persistent HR‐HPV infection had substantially higher odds of disease progression (adjusted OR: 14.9, 95% CI: 3.19–87.4) than those with persistent negative HR‐HPV results (Table S1). Conversely, persistent HR‐HPV infection was associated with lower odds of cervical disease regression compared to persistent negative HR‐HPV results (adjusted OR: 0.06, 95% CI: 0.01–0.37).

### Cervical disease transitions and natural history

**FIGURE 2:**
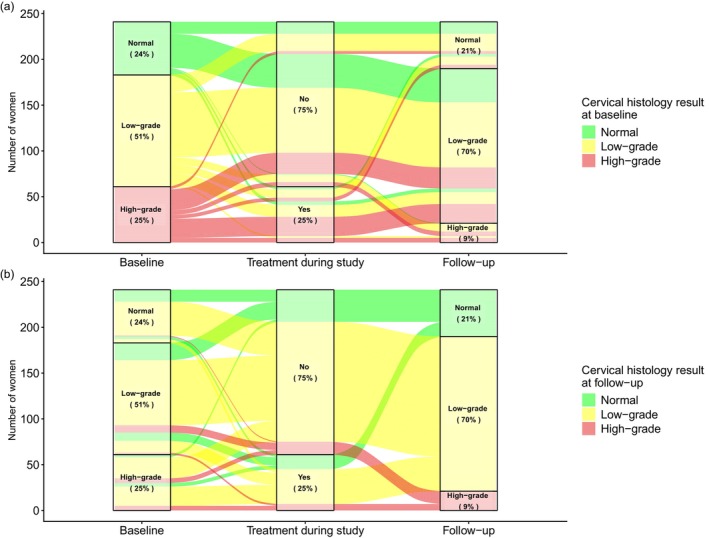
Temporal changes in cervical disease considering precancer treatment during the study period, with colours based on cervical disease status at baseline (panel a) or follow‐up (panel b).

Overall, 131 of the 241 included women (54%) experienced a change in disease state between baseline and follow‐up. Among 61 women with high‐grade disease at baseline, 72% (*n* = 44) experienced regression to low‐grade disease and 11% (*n* = 7) to normal histology (Figure 2a). Most of these regressions occurred in women who had either received treatment for HSIL‐CIN3 or HSIL‐CIN2 (with positive p16 staining) or had untreated HSIL‐CIN2 without p16 staining (Table 3). Ten women with high‐grade disease at baseline (16%) had persistent high‐grade disease at follow‐up. Among women with low‐grade disease at baseline, 69% remained in that state at follow‐up. For those with normal histology at baseline, 71% progressed to low‐grade disease. Figure 2b shows transitions in cervical disease states from baseline to follow‐up, with colours reflecting disease status at follow‐up. Most women with normal histology at follow‐up either had normal histology at baseline (31%) or had experienced regression from low‐grade disease without treatment (37%). Notably, seven of 21 women (33%) with high‐grade disease at follow‐up had previously undergone precancer treatment for high‐grade or low‐grade disease (Figure 2a). No invasive cervical cancer was detected at the follow‐up study visit.

**TABLE 3:** Distribution of histology results at follow‐up among women with high‐grade lesions at baseline, stratified by precancer treatment.

	Histology result at baseline					
	HSIL (*N* = 61)					
	CIN2 without p16^a^ (*N* = 20)		CIN2 with p16 (*N* = 25)		CIN3 (*N* = 16)	
	Untreated (*n* = 18)	Treated (*n* = 2)	Untreated (*n* = 9)	Treated (*n* = 16)	Untreated (*n* = 4)	Treated (*n* = 12)
Histology result at follow‐up						
Normal (*N* = 7)	2	0	1	3	0	1
LSIL‐CIN1 (*N* = 42)	14	2	4	11	3	8
LSIL‐CIN2 (*N* = 2)	0	0	2	0	0	0
HSIL‐CIN2 (*N* = 7)^b^	1	0	0	2	1	3
HSIL‐CIN3 (*N* = 3)	1	0	2	0	0	0

^a^ These HSIL CIN2 lacked p16 staining because they were categorized as LSIL by the primary pathologist but were categorized as HSIL–CIN2 on consensus review.

^b^ All HSIL‐CIN2 cases at follow‐up had p16 staining.

Table 4 shows the distribution of HR‐HPV dynamics among 180 women who did not undergo cervical precancer treatment between the baseline and the follow‐up visit. Of those, 102 women tested persistently negative for HR‐HPV, 33 women cleared their baseline HR‐HPV infection, 20 women acquired a new HR‐HPV infection and 25 women tested persistently positive for HR‐HPV. Persistently negative HR‐HPV results were common among women with normal histology at baseline, irrespective of whether they experienced progression or not. For those with low‐grade disease at baseline, persistently negative HR‐HPV results were common among those with disease regression, but 75% of women with disease progression had persistently positive HR‐HPV results. Most women with persistent high‐grade disease also had persistently positive HR‐HPV results.

**TABLE 4:** Distribution of high‐risk human papillomavirus (HR‐HPV) dynamics among 180 women who did not undergo precancer treatment, stratified by cervical disease status at baseline and by cervical disease regression, persistence and progression.

	Normal at baseline (*N* = 51)		Low‐grade disease at baseline (*N* = 98)			High‐grade disease at baseline (*N* = 31)	
	Persistence (*N* = 13)	Progression (*N* = 38)	Regression (*N* = 19)	Persistence (*N* = 71)	Progression (*N* = 8)	Regression (*N* = 26)	Persistence (*N* = 5)
HR‐HPV infection							
Persistent negative (−/−) (*N* = 102)	7 (54%)	23 (61%)	14 (74%)	41 (58%)	1 (13%)	15 (58%)	1 (20%)
Cleared Infection (+/−) (*N* = 33)	2 (15%)	5 (13%)	5 (26%)	16 (22%)	0	4 (15%)	1 (20%)
New infection (−/+) (*N* = 20)	3 (23%)	6 (16%)	0	7 (10%)	1 (13%)	3 (12%)	0
Persistent positive (+/+) (*N* = 25)	1 (8%)	4 (10%)	0	7 (10%)	6 (75%)	4 (15%)	3 (60%)

## DISCUSSION

In this cohort of screened WLWH in Zambia, HR‐HPV prevalence decreased from baseline to the 30–36‐month follow‐up visit, and more than half of the included women experienced a change in their cervical disease state on histology. Most women with high‐grade cervical disease at baseline who transitioned to low‐grade disease at follow‐up had either treated HSIL‐CIN3 or HSIL‐CIN2 with positive p16 results. Women with high‐grade cervical disease at baseline who transitioned to low‐grade disease without treatment were mostly women for whom histological assessments at baseline were discrepant between the two pathologists, and a consensus diagnosis of HSIL‐CIN2 was made in the absence of p16 results. One third of women with high‐grade disease at follow‐up had undergone cervical precancer treatment during the study period. Among untreated women, persistent HR‐HPV infection was strongly associated with increased odds of cervical disease progression and decreased odds of disease regression.

Findings from our study and others indicate that the prevalence of HR‐HPV among WLWH tends to decrease as they undergo repeat screening with treatment of cervical precancerous lesions. In a prospective cohort study of 237 WLWH in Botswana, where CIN2+ treatment was provided, HR‐HPV prevalence decreased from 28% to 20% over one year [16]. In the general population, a negative HR‐HPV test result after cervical precancer treatment is regarded as a reliable test of cure with a negative predictive value of close to 100% based on a 2017 meta‐analysis [17]. However, this may not necessarily apply to WLWH. A randomized trial in Zambia with an HIV prevalence of almost 60% among the included women found that only about half of all HR‐HPV‐positive women who underwent precancer treatment were HR‐HPV‐negative at the 12‐month follow‐up visit [18]. Similarly, a trial among WLWH from Kenya estimated type‐specific clearance of any HR‐HPV to occur among 51% of women who underwent LEEP and only 39% who underwent cryotherapy [19]. The clinical relevance of a positive HR‐HPV test after cervical precancer treatment is not fully understood, but high cervical precancer treatment failure rates of approximately 20% among WLWH have been documented in a 2019 meta‐analysis [12]. Nevertheless, cervical precancer treatment plays a crucial role in disease management, and WLWH who underwent pre‐cancer treatment during our study period had 3‐fold increased odds of disease regression compared with untreated women.

The proportion of women in our study who experienced regression from high‐grade disease is substantially higher than the 24‐month pooled regression rate of 50% reported by a systematic review of CIN2 regression among non‐pregnant HIV‐negative women under active surveillance [20]. In our study, most regressions from high‐grade disease occurred either among women with treated HSIL‐CIN3 or p‐16‐positive HSIL‐CIN2 or among women with untreated HSIL‐CIN2 that lacked p16 staining. If some of these HSIL‐CIN2 without p16 staining had wrongly been classified as high‐grade disease, we may have overestimated the proportion of high‐grade disease that spontaneously regressed. Interestingly, in a US‐based study among 104 WLWH with CIN2, the regression proportion was higher among untreated (73%) than treated (43%) women [21]. Another small study among 80 WLWH with high‐grade cervical disease found 8% of spontaneous regression to normal and 39% to low‐grade cervical disease [22]. Spontaneous regression of HSIL‐CIN3 remains a subject of ongoing controversy, with the reported regression rates among women without HIV ranging from 1% to 38% [23]. Well‐controlled HIV disease might increase the chance of spontaneous regression of cervical disease, with ART use being associated with almost 3‐fold higher odds of spontaneous cervical disease regression among South African WLWH [24]. In contrast, lower CD4 cell counts [25] and high HIV RNA viral loads [4] have been associated with increased cervical disease incidence and progression. While we did not find a strong association of cervical disease progression with CD4 cell count, detectable HIV RNA viral loads tended to increase the odds of cervical disease progression and lower the odds of disease regression among WLWH on ART in our analysis. Further research among larger cohorts of WLWH is needed to better understand factors that might be associated with and potentially modify cervical disease progression and regression in this population.

Novel, objective biomarkers are needed to distinguish persistent high‐grade lesions from those that may regress. In our study, HR‐HPV infection was strongly associated with disease progression, with persistent HR‐HPV emerging as a particularly strong predictor of cervical disease progression among untreated WLWH. Conversely, we found that women with low‐grade or high‐grade disease were more likely to experience regression if they consistently tested negative for HR‐HPV. A study among WLWH in Burkina Faso and South Africa reported an 8‐fold increased risk of incident CIN2+ among women with persistent HR‐HPV compared to those who cleared their infection or were HR‐HPV‐negative at baseline [26]. However, additional biomarkers such as testing for DNA methylation [27] and HPV E6/E7 mRNA [28] may be required to help reduce unnecessary follow‐up and treatment. We also observed women who had disease progression despite testing persistently negative for HR‐HPV. Possible reasons include imperfect HPV test sensitivity, especially among WLWH or if the HR‐HPV viral load was low [29], cervical sampling variability, reactivation of latent HR‐HPV infections between study visits [30], or other factors such as genetic predisposition to cervical disease progression.

Our study adds to the growing body of research on HR‐HPV prevalence and the patterns of cervical disease progression and regression among WLWH in sub‐Saharan Africa [16, 24, 26, 31, 32, 33]. One of the strengths of our study was that we defined cervical disease by histological rather than cytological assessment. Furthermore, we collected cervical biopsies from all women at baseline and follow‐up to reduce misclassification of cervical disease. Our study also had limitations. At baseline, some samples classified as HSIL‐CIN2 on consensus had not undergone p16 staining because they had initially been categorized as normal or LSIL at the laboratory where p16 staining was performed. It is possible that some of these HSIL‐CIN2 were assigned a higher histological grade than warranted, which would have resulted in an overestimation of high‐grade disease at baseline and spontaneous regression during the study. However, in a sensitivity analysis, where we reclassified HSIL‐CIN2 without p16 staining as low‐grade disease, our results remained broadly similar. Also, despite substantial efforts, including home visits, only about two‐thirds of the 375 women who had participated in the screening test accuracy returned for a follow‐up visit after 30–36 months. This was mainly due to challenges in contacting them, as some women had relocated, or their addresses could not be found. The relatively small sample size limited the precision of our results and precluded additional analyses, such as formally investigating effect modification in the regression analysis. Moreover, our findings among screened WLWH may not be generalizable to all Zambian WLWH since cervical cancer screening coverage remains low in Zambia [34]. Finally, several women who required precancer treatment based on the assessment at the baseline visit did not undergo treatment despite extensive efforts by the study team. However, by describing disease progression and regression in women who did not undergo treatment, we gained insights resembling the natural history of HR‐HPV and cervical disease.

In conclusion, our study highlights that cervical screening and precancer treatment are key to reducing cervical disease progression among WLWH and ultimately achieving cervical cancer elimination, but efforts to improve treatment effectiveness among WLWH must be balanced with the risk of overtreatment.

## AUTHOR CONTRIBUTIONS

KT, PB, NL, AM and ER conceptualized the study. KT, PB, NL, AM and ER were involved in funding acquisition. MM and AM were involved in project administration and implementation. TM and MHM were involved in laboratory analyses or clinical care for participants. JA performed the data analysis. JA and ER wrote the first draft of the manuscript with input from KT. All authors were involved in the interpretation of the results, reviewed the manuscript and agreed with the final version.

## CONFLICT OF INTEREST STATEMENT

The authors declare no conflicts of interest.

## ETHICS STATEMENT

Ethical approval was granted by the National Health Research Authority and the University of Zambia Biomedical Research Ethics Committee (ref: 014‐09‐18), the Zambia Medicines Regulatory Authority (ref: DMS/7/9/22/CT/084), the International Agency for Research on Cancer (IEC project number 18‐15) and the Cantonal Ethics Committee Bern (ref:PB_2016‐00273). This study involves human participants, all of whom provided informed consent prior to participation.

## Supporting information


**Table S1.** Crude and adjusted odds ratios (ORs) for factors potentially associated with cervical disease progression and regression among women who did not undergo precancer treatment.
**Table S2.** Adjusted odds ratios (ORs) for factors potentially associated with cervical disease progression and regression from a sensitivity analysis with HSIL‐CIN2 without p16 staining reclassified as low‐grade disease.
**Figure S1.** Flow diagram showing the selection of study participants.

## Data Availability

Due to ethical and legal restrictions related to participant privacy, the data underlying this study cannot be made publicly available. The data may be shared upon reasonable request to the corresponding author, subject to appropriate institutional and ethics approvals.
