# Supplementary material for: Cervical intraepithelial neoplasia progression and regression among women living with HIV in Zambia
Source: HIV Med. 2025 Nov 5;26(12):1939–49. doi: 10.1111/hiv.70134 (PMC12666240; doi:10.1111/hiv.70134)

**Supplementary file: Cervical intraepithelial neoplasia progression and regression among women living with HIV in Zambia**

**Table S1:** Crude and adjusted odds ratios (ORs) for factors potentially associated with cervical disease progression and regression among women who did not undergo precancer treatment.

|  | **Progression among 149 women with normal/low-grade cervical disease at baseline** | | | | **Regression among 129 women with low-grade or high-grade cervical disease at baseline** | | | |
| --- | --- | --- | --- | --- | --- | --- | --- | --- |
|  | **Progression**  **(N=46)** | **No progression (N=103)** | **Crude OR**  **(95% CI)** | **Adjusted OR**  **(95% CI)** | **Regression (N=45)** | **No regression (N=84)** | **Crude OR**  **(95% CI)** | **Adjusted OR**  **(95% CI)** |
| **Age (per 10-year increase)** | - | - | 1.06 (0.70, 1.61) | 1.10 (0.59, 2.10) | - | - | 0.94 (0.63, 1.39) | 1.08 (0.63, 1.87) |
| **HIV CD4 cell count**  **(per 100 increase)** | - | - | 1.03 (0.90, 1.19) | 1.16 (0.95, 1.45) | - | - | 0.94 (0.80, 1.09) | 1.01 (0.80, 1.26) |
| Missing | 1 | 0 | - | - | - | - | - | - |
| **HIV RNA viral load** |  |  |  |  |  |  |  |  |
| Undetectable | 40 (87%) | 94 (91%) | Ref | Ref | 42 (93%) | 76 (90%) | Ref | Ref |
| Detectable | 6 (13%) | 9 (9%) | 1.57 (0.49, 4.64) | 1.52 (0.32, 7.92) | 3 (7%) | 8 (10%) | 0.68 (0.14, 2.48) | 1.15 (0.15, 6.72) |
| **High-risk HPV infection** |  |  |  |  |  |  |  |  |
| Persistent negative (-/-) | 25 (54%) | 62 (60%) | Ref | Ref | 29 (64%) | 43 (51%) | Ref | Ref |
| Cleared infection (+/-) | 5 (11%) | 23 (22%) | 0.54 (0.17, 1.48) | 0.52 (0.09, 2.68) | 9 (20%) | 17 (20%) | 0.78 (0.30, 1.97) | 0.81 (0.23, 2.53) |
| New infection (-/+) | 6 (13%) | 10 (10%) | 1.48 (0.46, 4.46) | 0.63 (0.12, 3.56) | 3 (7%) | 8 (10%) | 0.56 (0.11, 2.10) | 0.23 (0.02, 1.66) |
| Persistent positive (+/+) | 10 (22%) | 8 (8%) | 3.10 (1.10, 9.02) | 14.9 (3.19, 87.4) | 4 (9%) | 16 (19%) | 0.37 (0.10, 1.13) | 0.06 (0.01, 0.37) |
| **Cervical histology at baseline** |  |  |  |  |  |  |  |  |
| Normal | 38 (83%) | 13 (13%) | 32.9 (13.2, 91.3) | 78.4 (23.4, 373) | - | - | - | - |
| Low-grade disease | 8 (17%) | 90 (88%) | Ref | Ref | 19 (42%) | 79 (94%) | Ref | Ref |
| High-grade disease | - | - | - | - | 26 (58%) | 5 (6%) | 21.6 (7.89, 70.8) | 56.9 (14.1, 428) |

**Table S2:** Adjusted odds ratios (ORs) for factors potentially associated with cervical disease progression and regression from a sensitivity analysis with HSIL-CIN2 without p16 staining reclassified as low-grade disease.

|  | **Progression among 201 women with normal/low-grade cervical disease at baseline** | | | **Regression among 183 women with low-grade or high-grade cervical disease at baseline** | | |
| --- | --- | --- | --- | --- | --- | --- |
|  | **Progression**  **(N=54)** | **No progression (N=147)** | **Adjusted OR**  **(95% CI)** | **Regression (N=62)** | **No regression (N=121)** | **Adjusted OR**  **(95% CI)** |
| **Age (per 10-year increase)** | - | - | 1.40  (0.83, 2.39) | - | - | 0.76  (0.50, 1.14) |
| **HIV CD4 (per 100 increase)** | - | - | 1.14  (0.96, 1.35) | - | - | 0.98  (0.84, 1.13) |
| **HIV RNA viral load** |  |  |  |  |  |  |
| Undetectable | 47 (87%) | 133 (91%) | Ref | 54 (87%) | 107 (88%) | Ref |
| Detectable | 7 (13%) | 14 (9%) | 2.48  (0.64, 9.65) | 8 (13%) | 14 (12%) | 0.57  (0.14, 1.91) |
| **High-risk HPV infection at baseline** |  |  |  |  |  |  |
| No | 32 (62%) | 95 (65%) | Ref | 24 (39%) | 68 (56%) | Ref |
| Yes | 22 (38%) | 52 (35%) | 4.61  (1.69, 14.28) | 38 (61%) | 53 (44%) | 0.67  (0.27, 1.57) |
| **Cervical histology at baseline** |  |  |  |  |  |  |
| Normal | 42 (88%) | 16 (11%) | 48.2  (18.6,148.5) | - | - | - |
| Low-grade disease* | 12 (22%) | 131 (89%) | Ref | 30 (48%) | 113 (93%) | Ref |
|  |  |  |  |  |  |  |
| High-grade disease | - | - | - | 32 (52%) | 8 (7%) | 12.78  (4.98, 36.37) |
| **Precancer treatment** |  |  |  |  |  |  |
| No | 48 (89%) | 120 (82%) | Ref | 30 (48%) | 99 (82%) | Ref |
| Yes | 6 (11%) | 27 (18%) | 0.37  (0.09, 1.29) | 32 (52%) | 22 (18%) | 2.52  (1.02, 6.24) |

*women with CIN2 on histology but lacked p16 results were considered as having low-grade disease

**Figure S1:** Flow diagram showing the selection of study participants.


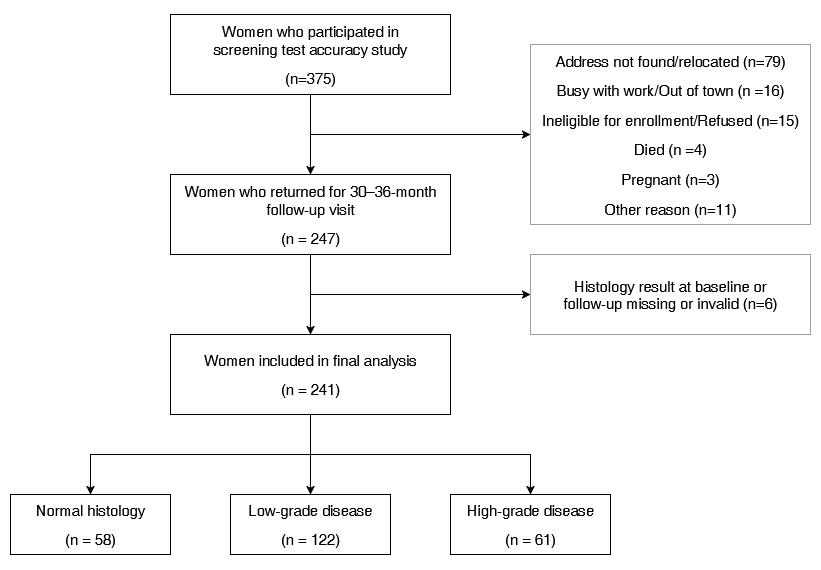

Supplement: Supplementary file 1 — Table S1. Crude and adjusted odds ratios (ORs) for factors potentially associated with cervical disease progression and regression among women who did not undergo precancer treatment. Table S2. Adjusted odds ratios (ORs) for factors potentially associated with cervical disease progression and regression from a sensitivity analysis with HSIL‐CIN2 without p16 staining reclassified as low‐grade disease. Figure S1. Flow diagram showing the selection of study participants. [file HIV-26-1939-s001.docx]
